# Flavone Derivatives as Inhibitors of Insulin Amyloid-Like Fibril Formation

**DOI:** 10.1371/journal.pone.0121231

**Published:** 2015-03-23

**Authors:** Ricardas Malisauskas, Akvile Botyriute, Jonathan G. Cannon, Vytautas Smirnovas

**Affiliations:** 1 Department of Biothermodynamics and Drug Design, Vilnius University Institute of Biotechnology, Vilnius, Lithuania; 2 Department of Chemistry and Bioengineering, Vilnius Gediminas Technical University, Vilnius, Lithuania; 3 Department of Natural Sciences and Engineering, Middle Georgia State College, Cochran, Georgia, USA; University of Maryland School of Medicine, UNITED STATES

## Abstract

Several natural and synthetic flavone derivatives have been reported to inhibit formation of amyloid fibrils or to remodel existing fibrils. These studies suggest that the numbers and positions of hydroxyl groups on the flavone rings determine their effectiveness as amyloid inhibitors. In many studies the primary method for determining the effectiveness of inhibition is measuring Thioflavin T (ThT) fluorescence. This method demonstrably results in a number of false positives for inhibition. We studied the effects of 265 commercially available flavone derivatives on insulin fibril formation. We enhanced the effectiveness of ThT fluorescence measurements by fitting kinetic curves to obtain halftime of aggregation (*t*_50_). Maximal values of ThT fluorescence varied two fold or more in one third of all cases, but this did not correlate with changes in *t*_50_. Changes in *t*_50_ values were more accurate measures of inhibition of amyloid formation. We showed that without a change in an assay, but just by observing complete kinetic curves it is possible to eliminate numbers of false positive and sometimes even false negative results. Examining the data from all 265 flavones we confirmed previous observations that identified the importance of hydroxyl groups for inhibition. Our evidence suggests the importance of hydroxyl groups at locations 5, 6, 7, and 4’, and the absence of a hydroxyl group at location 3, for inhibiting amyloid formation. However, the main conclusion is that the positions are not additive. The structures and their effects must be thought of in the context of the whole molecule.

## Introduction

Alzheimer’s disease, Parkinson’s disease, Huntington’s disease, transmissible spongiform encephalopathies, familial amyloid neuropathy, and diabetes are among the diseases associated with formation of amyloid fibrils. Both mature amyloid fibrils, and oligomers or protofibrils which can exist on pathway of fibril formation, may be responsible for pathogenesis, depending on the disease. Small molecules able to change kinetics or alter the pathway of protein aggregation are of interest to treat or prevent these diseases. A number of compounds have been reported to inhibit amyloid fibril formation. Some of these, such as epi-gallocatechine-3-gallate (EGCG) can even remodel mature fibrils into non-toxic aggregates [1]. In cases where oligomeric intermediates are responsible for the pathology, compounds which accelerate fibril formation may also prove useful in treatment [2].

Several flavone derivatives have been reported to inhibit fibril formation of different proteins and peptides. Ono et al. found that certain flavone derivatives and related compounds inhibited and/or destabilized amyloid beta (A*β*) fibrils [3]. Kim et al. tested a large number of small molecules for inhibition of thioflavin T (ThT) fluorescence in A*β* fibril formation and for protection of neuroblastoma cells against the effects of A*β* fibril induced oxidative stress [4]. While numerous flavones reduced ThT fluorescence, none protected neuroblastoma cells against oxidative stress. Akaishi et al. used ThT fluorescence to test the effectiveness of ten flavonoids in A*β* fibril formation, and from this concluded the importance of hydroxyl substituents at particular locations for fibril inhibition [5]. Sharoar et al. used numerous methods to show that a flavone-rhamnoside was effective at preventing A*β* fibrillation or remodelling A*β* fibrils into non-toxic oligomers [6]. Ushikubo et al. used ThT fluorescence to test several synthesized flavonoids for inhibition and remodelling of A*β* fibrils. They also used electron microscopy to confirm morphological changes for representative experiments [7]. Similar kinds of studies have examined the effects of flavonoids on other amyloid forming proteins. Green et al [8] and Trivella et al. [9] studying transthyretin (TTR) report effects of only a few flavone derivatives, but employed a wide variety of methods to present a quite detailed description of their effects. Unlike with A*β*, a significant contribution to fibril inhibition is from interactions of the flavonoids with native TTR tetramers. Fibrillation inhibition by hydroxyflavones has also been studied for islet amyloid polypeptide (IAPP) [10]. Noor et al. employed ThT fluorescence, kinetic measurements, electron microscopy, and light scattering to learn as much as possible about the effects of four flavonoids. Borana et al. added molecular dynamics simulations and binding energy calculations to multiple biochemical studies of the effects of two flavone derivatives on lysozyme fibril formation [11], but could only simulate interactions with the native monomers. One flavonoid has been studied in some depth as it affects bovine insulin amyloid formation [12]. In addition to morphological characterization of amyloid and other aggregates, protection of erythrocytes from hemolysis was measured and found to be dose dependent.

A few generalizations and a number of questions arise from surveying these various studies. Hydroxyflavones can inhibit fibril formation with many amyloid forming proteins. Different flavonoids may affect different amyloid reactions. Investigating any one inhibitor thoroughly is both labor and resource intensive, and the easiest method for screening large numbers of small molecules is observing ThT fluorescence—a technique employed by nearly every study. It may be possible to design better inhibitors by optimizing side groups of flavones, as suggested for A*β* [7], but we need to emphasize again the difficulty and time required to study a single inhibitor thoroughly, performing both biophysical studies of fibril formation and morphology and biochemical, inhibition of toxicity studies. An improved screening method for small molecule inhibitors of fibril formation could be valuable to direct future studies toward greater focus and productivity.

We selected insulin as an initial model for amyloid-like fibril formation to demonstrate our improved screening methodology. Insulin is relatively inexpensive and forms amyloid under a variety of conditions [13–15]. Formation of insulin amyloid-like deposits has also been reported in several cases of injection-localized amyloidosis [16–18] among diabetics. We found 265 commercially available flavone derivatives to test as inhibitors of insulin amyloid formation.

We used the nearly universal thioflavin T fluorescence assay, but collected and analysed kinetic data as an additional check for amyloid formation. A number of studies have evaluated the ability of compounds to inhibit or accelerate fibril formation based primarily on the decrease or increase of ThT fluorescence intensity in the presence of presumed fibrils [3–7, 12]. Several reports have shown reasons to use additional techniques to confirm the results of ThT assays because pH, time, temperature, and other small molecules can all interfere with the ThT fluorescence, thus biasing results [19–21]. In particular, Noormagi et al showed that upon addition of some compounds (Basic Blue 41, Basic Blue 12, Azure C, or Tannic acid) to preformed insulin fibrils, ThT fluorescence intensity strongly decreases, however neither lag time, nor the rate constant is affected by these compounds; it was concluded that most probably these compounds compete with ThT for the same binding sites in fibrils [20]. Hudson et al showed that Quercetin (one of the flavones we also used in our study) and Curcumin decreases ThT fluorescence in a concentration-dependent manner when added to A*β* fibrils. They also showed that in case of reduced and carboxymethylated kappa-casein fibrils, Quercetin acts the same as with A*β*, however concentrations of Curcumin up to 10 *μ*M can increase ThT fluorescence, but higher concentrations quench it. The study concludes that spectroscopic effects are the prominent contributor to the interference with ThT fluorescence by these two polyphenols [19]. We show here that simply observing complete kinetic curves of protein aggregation in presence of flavones, and using aggregation halftimes as the main parameter for the determination of the influence of the compound eliminates at least 80% of false positives for amyloid inhibition resulting from uncorrelated decreases in ThT fluorescence. In this way, nearly 300 small molecules were tested for inhibitory effects and we were able to reduce the number of candidates for future study to just a handful.

## Materials and Methods

Recombinant human insulin was purchased from Sigma Aldrich, and flavones were purchased from Indofine Chemical Company. Flavones were dissolved at a concentration of 20 mM in dimethylsulfoxide (DMSO) and stored in the dark at room temperature for up to two weeks. Fresh 1.05 mM insulin solution in 100 mM phosphate buffer, pH2, containing 50 *μ*M ThT was mixed with flavone derivative stock solution (or with pure DMSO as a control) in ratio 19:1 leading to final concentrations of 1 mM insulin, 1 mM flavone derivative and 5% DMSO. Each batch was divided into three 20 *μ*l aliquots (in 200 *μ*l thin-wall PCR tubes). Aggregation kinetics was followed at constant 60°C temperature using Qiagen Rotor-Gene Q real-time analyser. Increase of ThT fluorescence intensity upon fibril formation was observed using the green channel (excitation 470 nm; emission 510 nm). To obtain *t*_50_ values we used the method, described by Nielsen et al [13]. According to nucleated growth mechanism, it takes time to form aggregation nuclei, which is reflected as initial lag phase with no change in ThT fluorescence intensity. Once formed, nuclei can recruit protein molecules and grow into fibrils, which is reflected as the growth phase in our kinetic curves. Finally, due to depletion of free protein molecules, the process enters into the final equilibrium phase, reflected in a plateau of ThT fluorescence. This type of kinetics can be fitted by a sigmoidal curve, described by the following equation [13]:

$$\begin{array}{c} I=I_{min}+m_{min}t+\frac{I_{max}+m_{max}t}{1+e^{-[\left(t-t_{50}\right)k_{app}]}} \end{array}$$ (1)

where *I* is the fluorescence intensity, *t* is time, and *t*_50_ is the time to 50% of maximal fluorescence, *I*_*min*_ and *I*_*max*_ are values of minimal and maximal fluorescence intensities, respectively, *m*_*min*_ *t* and *m*_*max*_ *t* describe drift of the baseline at the beginning and at the end of the reaction respectively. *k*_*app*_ is the apparent rate constant. Observed *t*_50_ and *I*_*max*_ values in presence of flavone derivatives were divided by the observed values for the control samples to obtain relative *t*_50_ and *I*_*max*_. Fitting was performed using MathCad software. Average values and errors were calculated using three different batch preparations of flavones and three samples within each batch (a total of 9 repeats per flavone derivative). See an example of raw data and fitting in S1 Fig and S2 Fig.

To check possible impact of ThT on fibrillation kinetics insulin samples with or without flavones in presence or absence of 50 *μ*M ThT were prepared as described above. Samples were divided into 200 *μ*l aliquots, in 96-well plates. Plates were sealed using clear polyolefin sealing tape. Aggregation kinetics was followed at constant 60°C temperature using Biotek Synergy H4 plate reader. ThT fluorescence intensity upon fibril formation was observed using 440 nm excitation and 480 nm emission with simultaneous measurement of absorbance at 600 nm. The data confirmed no interference by ThT of the inhibition by flavones (S3 Fig).

Seeding experiments were performed as described earlier [22]. Briefly, seeds were prepared by incubation of 1mM insulin solution (100mM phosphate, pH2) at 60°C for 24 hours with 300 rpm agitation and subsequent ultrasonic treatment. One part of the fibrils were mixed with 9 parts of the fresh 1 mM insulin solution containing 50 mM Thioflavin T, 1 mM of tested flavone, and 5% residual DMSO. Elongation kinetics was measured at constant 37°C temperature using Qiagen Rotor-Gene Q real-time analyzer. Rates of elongation in the absence and presence of flavones were determined by linear fits to the curves in a range between 40–60% of the ordinate maxima, as described earlier [23]. Observed rate (*v*) values in presence of flavone derivatives were divided by the observed values for the control samples to obtain relative *v*.

## Results and Discussion

**Fig 1:**
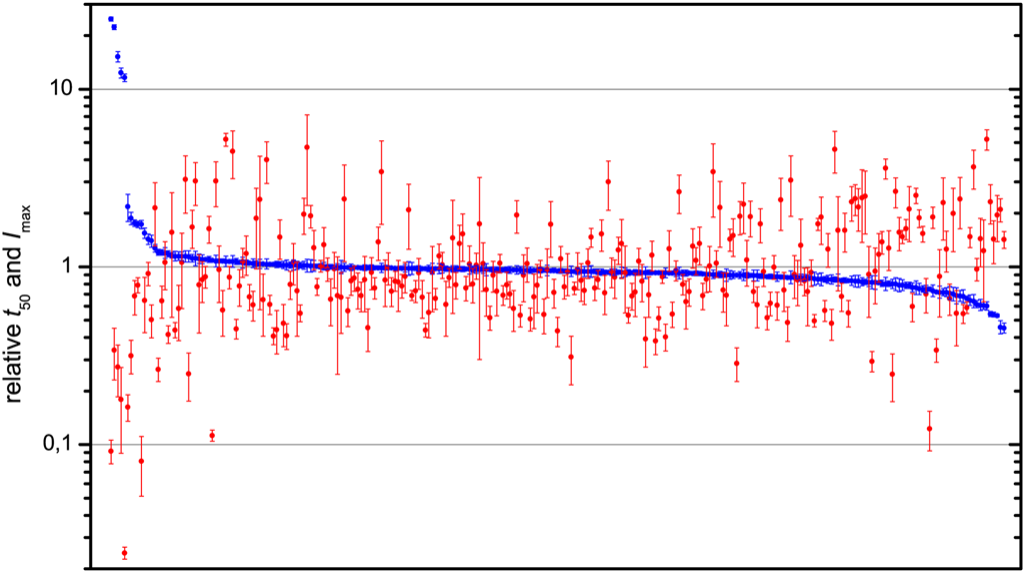
Evaluation of flavones as inhibitors of insulin amyloid-like fibril formation. Blue dots represent relative *t*_50_ values for fibril formation in the presence of each flavonoid. Red dots represent relative *I*_*max*_ of the corresponding samples (see complete list of names and values in S1 Table). Error bars represent standard errors, calculated using normal distribution with P = 0.05.

Fig. 1 summarizes how all 265 flavone derivatives influence insulin fibril formation. Changes in *t*_50_ relative to control samples are shown in blue with the best inhibitors on the left moving across to flavonoids which enhance amyloid formation at the right. Most flavone derivatives have no impact on time of aggregation, with 243 (92%) of the flavonoids giving relative values of *t*_50_ between 1.50 and 0.67, and only 8 changing *t*_50_ by more than a factor of two. By contrast, maximal ThT fluorescence varies over a wide range and correlates very weakly with fibrillation kinetics. 33 of the flavonoids decreased maximum ThT fluorescence by more than a factor of two, and 36 increased fluorescence two fold or more, despite the majority of these having minimal impact on fibrillation. The best inhibitors showed both increases in aggregation halftimes and decreases in ThT intensity. However, relying solely on the ThT fluorescence would have resulted in 10% false positives for amyloid inhibition, which is significant considering only 2% of flavonoids significantly inhibited fibrillation. Similar problems are evident for identifying fibrillation accelerators.

It is known that the rate of amyloid fibril formation depends on protein concentration and the concentration of nucleation sites on existing fibrils [24, 25]. If ThT fluorescence intensity is interpreted as the concentration of amyloid-like fibrils, lower fluorescence means a portion of the protein is kept away from the fibrillation pathway, so the concentration of nucleating fibrils and the concentration of monomers available for fibrillation are both lower. Thus, aggregation time should increase. No change in *t*_50_ means that changes in ThT fluorescence are caused by factors other than concentration of amyloid, such as interference from the flavonoids [19, 20].

**Fig 2:**
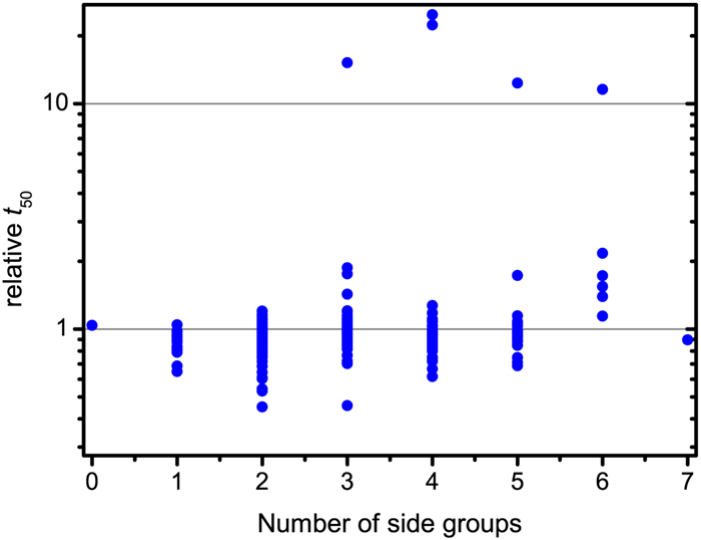
Distribution of inhibition potential by the number of side groups.

Beyond testing ThT fluorescence we looked for patterns in the effects of substituent groups on the flavone rings in inhibiting fibrillation. Aggregation time dependence on the number of substituents is shown in Fig. 2. Flavone without any side groups shows no impact on the rate of fibril formation. As noted above, the majority of flavones, no matter how many substituent groups, have little effect on fibrillation rates. We now note the outliers. No flavones with one or two side groups inhibit fibrillation. Most of the best fibrillation accelerators have two side groups, while one has three substituents. The first strong inhibitor, 7,8,2’-trihydroxyflavone, also has three side groups. The tetra-substituted flavones include the two strongest inhibitors, Scutellarein and Luteolin. Penta-substituted flavones include one medium and one strong inhibitor (3,6,2’,4’,5’-Pentahydroxyflavone). All hexahydroxyflavones tested show some inhibition, and one of these is a strong inhibitor (Gossypetin). However, we had only six such flavones available, so it is impossible to make strong generalizations. Overall we can state that flavones with two or fewer hydroxyl groups have no inhibition potential. The best inhibitors yet measured are tetra-substituted flavones, though there are also good inhibitors among tri-, penta-, and hexa-substituted flavones. Such distribution means that three residues around the flavone backbone are enough to have a potent inhibitor, however, the inhibition potential can be both increased and decreased by additional residues.

Among the commercially available flavones the majority contain hydroxyl and methoxy groups. There are also some flavones containing chloride, bromide, or methyl groups, some flavone glucosides, and naphtoflavones. Surprisingly, no flavone containing substituents other than hydroxyl and methoxy side groups inhibited insulin amyloid-like fibril formation. For some chemical groups our data may be inconclusive due to the low numbers of representative compounds, however in the cases of bromo- (20 compounds), chloro- (41 compounds), and methyl- (35 compounds) flavones it may be significant.

**Fig 3:**
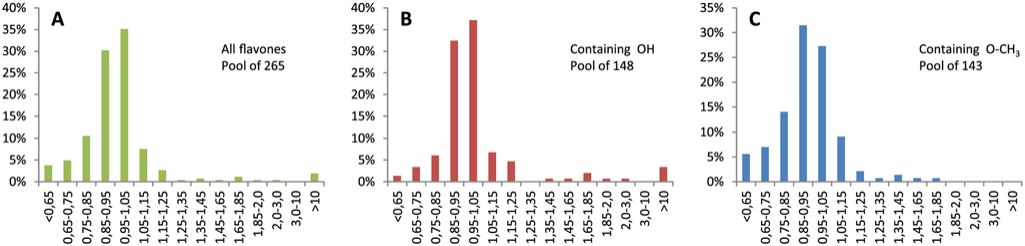
Distribution of relative *t*_50_ among flavones. All tested flavones (A), flavones with at least one hydroxyl group (B), and with at least one methoxy group (C). The height of the bars shows the percentage of total flavones in each range of relative *t*_50_ values.

Fig. 3A shows distribution of relative halftimes of aggregation among all tested flavones. Two thirds of the compounds have no impact or slightly accelerate the fibril formation. Almost 20% of tested flavones moderately accelerate fibrillation, and only about 5% are medium or strong inhibitors. Flavones containing at least one hydroxyl group have a similar distribution profile to all flavones (Fig. 3B), but include all of the medium and strong inhibitors (∼8%) and a reduced fraction of accelerators (∼11%). In case of methoxy groups, the profile is shifted towards acceleration of aggregation (Fig. 3C). More than 25% of the medium accelerators and only a few moderate inhibitors contain methoxy groups. This leads to the conclusion that, among the tested side groups, only hydroxyl groups can increase the ability of flavones to inhibit amyloid-like fibril formation.

**Fig 4:**
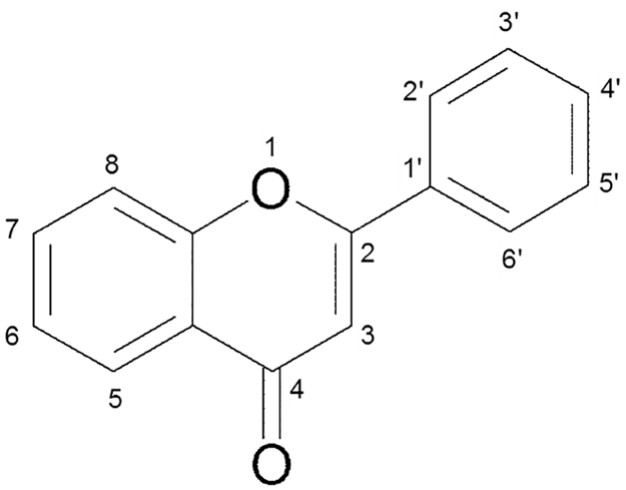
Flavone backbone with numbered positions for residues.

**Fig 5:**
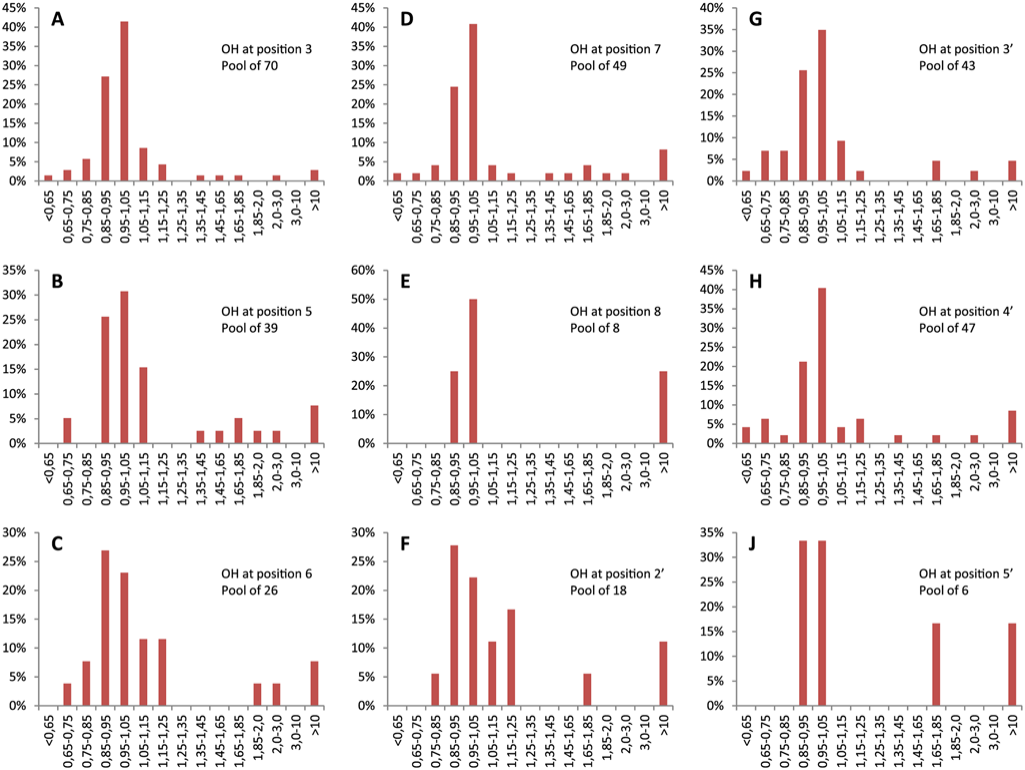
Influence of hydroxyl residue position on the relative halftimes of aggregation.

To understand how the position of hydroxyl groups affects inhibition potential, we plotted relative *t*_50_ as a function of hydroxyl group position around the flavone backbone (Fig. 4). For discussion we will consider five ranges of relative *t*_50_ values: < 0.65 (moderate accelerator), 0.65–0.85 (weak accelerator), 0.85–1.15 (no effect), 1.15–3.0 (weak inhibitor), and > 10.0 (strong inhibitor). More divisions are retained in Fig. 5 to give a more complete account of the distribution of effects from flavones on amyloid formation. Fig. 5 shows the observed effects of hydroxyl groups at all of the nine positions available for substitution without having a penta-substituted phenyl group. Out of the 265 flavones studied, there are 10 moderate accelerators, 41 weak accelerators, 16 weak inhibitors, and 5 strong inhibitors. One thing made clear by our groupings is that no flavones resulted in relative *t*_50_ between 3 and 10. This leads to our first observation—hydroxyl groups do not simply have an additive effect, but must interact cooperatively with insulin or insulin aggregates. Despite this clear cooperativity, we looked to see if any single positions could be identified as essential or important to inhibition or acceleration.

The most common position for a hydroxyl group among commercially available flavone derivatives is 3 (Fig. 5A). Predictably, the majority of this group of flavone derivatives have no effect on *t*_50_, but 2 of the 5 strong inhibitors, and 1 of 10 moderate accelerators are included in this group. A hydroxyl at position 5 (Fig. 5B) is present in 3 of 5 strong and 6 of 16 weak inhibitors, and only in 2 of 41 weak accelerators. Position 6 (Fig. 5C) is similar to 5 in that 2 of 5 strong and 5 of 16 weak inhibitors have hydroxyl groups at this position, while only 3 of 41 weak accelerators have hydroxyls at 6. Hydroxyl at position 7 (Fig. 5D) is present in 4 of 5 strong inhibitors, as is hydroxyl at position 4’ (Fig. 5H), but hydroxyls are also present at these positions in the only moderate accelerator containing four hydroxyl groups. Few flavones had hydroxyl groups at 8, 2’, and 5’ (Fig. 5E, F, and J), but hydroxyl groups were found in each of these positions in 1 or 2 of the strong inhibitors. Also, hydroxyl groups were not at these positions in any of the accelerators, but with such small sample sizes that is not very informative. A hydroxyl at 3’ is also ambiguous, being present in 2 strong inhibitors and 1 moderate accelerator. Other than the general trend that more hydroxyl groups are more likely to result in inhibition of amyloid formation, little can be said about hydroxyl groups in specific positions removed from the context of the rest of the molecule.

Despite the recognized ambiguities, we have tested enough flavone derivatives with hydroxyl groups at certain positions to suggest positions which may be more or less likely to increase *t*_50_. We determined the numbers of possible mono- to hexa-substituted hydroxyflavonoinds, including a group at each given position (e.g. 236 possible different 3-, mono- to hexa- hydroxyflavone derivatives). We then used the number of hydroxyflavonoids tested to calculate a percentage of the total, as shown in Table 1. With 11% of all possible 3-hydroxyflavone derivatives tested, 3 appears to be a weak position for favouring either inhibition or acceleration. 9–14% of all possible 5-, 6-, 7-, 3’-, or 4’- hydroxyflavone derivatives were tested, revealing 5, 6 and 7 as more likely positions for favouring inhibition, 3’ as less likely to favour inhibition, and 4’ as potentially the most likely to contribute to either inhibition or acceleration and not simply have a neutral effect. Insufficient numbers of other hydroxyflavone derivatives were tested to even consider general statistical assertions.

**Table 1:** Number of hydroxyflavones with a hydroxyl group at a given position.

Position	total flavones*	tested flavones	% tested
3	236	27	11
5	236	21	9
6	236	21	9
7	236	34	14
8	236	7	3
2’	340	16	5
3’	283	30	11
4’	236	32	14
5’	178	6	3

*Only flavones with 1–6 hydroxyl groups were included.

To examine the effects of hydroxyl groups in context, we compared the five strongest inhibitors with the other most structurally similar compounds. One of the best inhibitors is 5,7,3’,4’-tetrahydroxyflavone (also known as Luteolin). There were 12 similar flavones in our pool. One of these is among five strongest inhibitors, however none of the rest showed any inhibition potential (Table 2). It looks like either removal of a hydroxyl group at 5,7 or 3’, or addition of a hydroxyl group at 3 or 5’ leads to the complete loss of inhibition. Exchange of a hydroxyl from position 5 to positions 3, 6, 8, or 5’ also results in the loss of inhibition. Moreover, two of these rearranged tetrahydroxyflavones accelerate insulin fibril formation. The only exchange without a loss of inhibition is from position 3’ to 6.

**Table 2:** Comparison of inhibition potentials of Luteolin and similar flavones.

Compound	Substituents									*t*_50_
	3	5	6	7	8	2’	3’	4’	5’	
Luteolin	H	OH	H	OH	H	H	OH	OH	H	22.3 ± 0.7
Quercetin	**OH**	OH	H	OH	H	H	OH	OH	H	1.03 ± 0.02
5,7,3’,4’,5’-Pentahydroxyflavone	H	OH	H	OH	H	H	OH	OH	**OH**	0.97 ± 0.05
7,3’,4’-Trihydroxyflavone	H	**H**	H	OH	H	H	OH	OH	H	0.98 ± 0.08
Apigenin	H	OH	H	OH	H	H	**H**	OH	H	0.96 ± 0.04
5,3’,4’-Trihydroxyflavone	H	OH	H	**H**	H	H	OH	OH	H	0.93 ± 0.02
Diosmetin	H	OH	H	OH	H	H	OH	**O-CH_3_**	H	0.90 ± 0.07
7,3’,4’,5’-Tetrahydroxyflavone	H	**H**	H	OH	H	H	OH	OH	**OH**	0.96 ± 0.05
7,8,3’,4’-Tetrahydroxyflavone	H	**H**	H	OH	**OH**	H	OH	OH	H	0.93 ± 0.07
6,7,3’,4’-Tetrahydroxyflavone	H	**H**	**OH**	OH	H	H	OH	OH	H	0.80 ± 0.05
Fisetin	**OH**	**H**	H	OH	H	H	OH	OH	H	0.62 ± 0.03
Kaempferol	**OH**	OH	H	OH	H	H	**H**	OH	H	0.98 ± 0.05
Scutellarein	H	OH	**OH**	OH	H	H	**H**	OH	H	24.8 ± 0.7

According to our data, the best inhibitor is 5,6,7,4’-tetrahydroxyflavone (also known as Scutellarein), providing a 25 fold increase in *t*_50_. Exchange of the hydroxyl from position 6 to position 3’ does not affect inhibition, however moving the hydroxyl group from 5 to 3’ leads to weak acceleration of fibrillation (Table 3). Exchange from 6 to 3 leads to loss of inhibitory activity. Loss of a hydroxyl group at position 6 eliminates inhibition; however, loss of the hydroxyl at position 4’ only reduces the relative *t*_50_ to ∼2.

**Table 3:** Comparison of inhibition potentials of Scutellarein and similar flavones.

Compound	Substituents									*t*_50_
	3	5	6	7	8	2’	3’	4’	5’	
Scutellarein	H	OH	OH	OH	H	H	H	OH	H	24.8 ± 0.7
Baicalein	H	OH	OH	OH	H	H	H	**H**	H	1.87 ± 0.15
Apigenin	H	OH	**H**	OH	H	H	H	OH	H	0.96 ± 0.04
6,7,3’,4’-Tetrahydroxyflavone	H	**H**	OH	OH	H	H	**OH**	OH	H	0.80 ± 0.05
Kaempferol	**OH**	OH	**H**	OH	H	H	H	OH	H	0.98 ± 0.05
Luteolin	H	OH	**H**	OH	H	H	**OH**	OH	H	22.3 ± 0.7

The only strong inhibitor with three side groups is 7,8,2’-trihydroxyflavone. Addition of a hydroxyl at position 3 or exchange of hydroxyl at position 2’ to 3’ or 4’ leads to no inhibition (Table 4). Exchange of position 8 to 5 leads to 10 fold weaker inhibition.

**Table 4:** Comparison of inhibition potentials of 7,8,2’-Trihydroxyflavone and similar flavones.

Compound	Substituents									*t*_50_
	3	5	6	7	8	2’	3’	4’	5’	
7,8,2’-Trihydroxyflavone	H	H	H	OH	OH	OH	H	H	H	15.2 ± 1.0
3,7,8,2’-Tetrahydroxyflavone	**OH**	H	H	OH	OH	OH	H	H	H	0.99 ± 0.02
5,7,2’-Trihydroxyflavone	H	**OH**	H	OH	**H**	OH	H	H	H	1.76 ± 0.07
7,8,3’-Trihydroxyflavone	H	H	H	OH	OH	**H**	**OH**	H	H	0.97 ± 0.03
7,8,4’-Trihydroxyflavone	H	H	H	OH	OH	**H**	H	**OH**	H	0.97 ± 0.04

Although all hexahydroxyflavones tend to inhibit insulin fibrillation, the only rather strong inhibitor is 3,5,7,8,3’,4’-hexahydroxyflavone (also known as Gossypetin). Loss of the hydroxyl group at position 8 or modification of it with a glucoside leads to loss of inhibition, whereas exchange of the position to 6 or 5’ only lowers inhibition (Table 5).

**Table 5:** Comparison of inhibition potentials of Gossypetin and similar flavones.

Compound	Substituents									*t*_50_
	3	5	6	7	8	2’	3’	4’	5’	
Gossypetin	OH	OH	H	OH	OH	H	OH	OH	H	11.6 ± 0.6
Quercetin	OH	OH	H	OH	**H**	H	OH	OH	H	1.03 ± 0.02
Quercetagetin	OH	OH	**OH**	OH	**H**	H	OH	OH	H	2.17 ± 0.38
Myricetin	OH	OH	H	OH	**H**	H	OH	OH	**OH**	1.73 ± 0.10
Gossypin	OH	OH	H	OH	**O-C_6_H_11_O_5_**	H	OH	OH	H	1.15 ± 0.08

For the last identified strong inhibitor, 3,6,2’,4’,5’-pentahydroxyflavone (*t*_50_ = 12.2 ± 0.8), there was only one very similar compound within our pool, 3,6,2’,4’-tetrahydroxyflavone (*t*_50_ = 1.18 ± 0.05). We can only note that the 5’ position is important in the specific interactions of this molecule.

**Fig 6:**
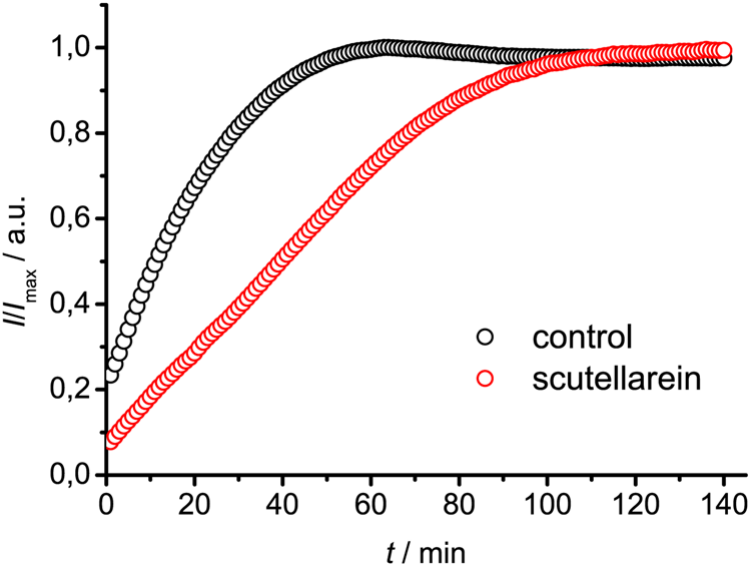
Impact of Scutellarein on insulin fibril elongation.

Nucleated growth mechanism of fibril formation assumes two main processes—nucleation and elongation. Relative *t*_50_ values obtained in our experiments are mostly influenced by changes in times of nucleation. To test if the rate of insulin fibril elongation can be slowed down, we did additional seeding experiments in presence of the five best-inhibiting flavones. Fig. 6 shows normalized curves of seeded insulin aggregation in the absence and presence of 1 mM Scutellarein. It clearly shows that Scutellarein inhibits fibril elongation; however it is not slowed down as many times as nucleation. In fact, calculated relative rates of elongation in the presence of Scutellarein, Luteolin, and 7,8,2’-trihydroxyflavone were the same (*v* = 0.44 ± 0.07), in the presence of Gossypetin aggregation was a bit slower (*v* = 0.40 ± 0.07), and 3,6,2’,4’,5’-pentahydroxyflavone was found to be the weakest inhibitor of elongation (*v* = 0.72 ± 0.13). In all cases nucleation was inhibited more strongly than elongation, suggesting that these flavones affect monomers or intermediates rather than mature fibrils.

**Fig 7:**
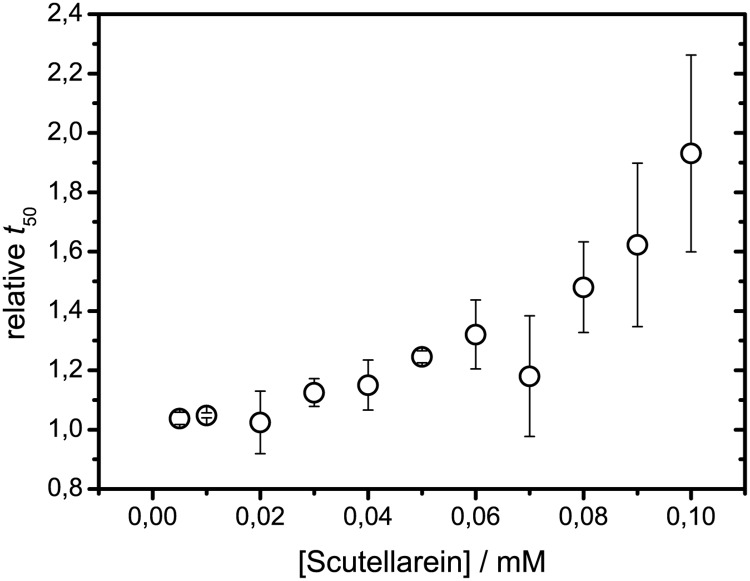
Dependence of relative aggregation times of insulin on Scutellarein concentration.

Additional experiments were done to check aggregation inhibition at different Scutellarein concentrations. As at equimolar concentration Scutellarein slows down spontaneous insulin aggregation almost 25 times, we decided to test if any inhibition can be detected in range of 0.01–0.1 mM (Insulin:Scutellarein ratios 100:1–10:1). Our data reveal that Scutellarein can make some impact even at very low concentrations and that the dependence of relative *t*_50_ *t*_50_ on concentration of the flavone is non-linear (Fig. 7). In this concentration range, relative *I*_*max*_ values are close to 1. It shows another advantage of analysing kinetics in screening for inhibitors of amyloid-like aggregation, as really strong inhibitors may be detected even at much smaller concentrations.

With these kinetic studies we have confirmed the need to corroborate ThT fluorescence measurements with results from other methods, demonstrated that multiple hydroxyl groups are essential for flavone derivatives to inhibit insulin amyloid formation, discovered that the effects of hydroxyl groups at specific positions are not additive, and suggested that hydroxyl groups at certain positions may be more likely to contribute to inhibition than at others. We did find that all of the strong inhibitors of fibrillation also reduced ThT fluorescence, but among the approximately 250 remaining flavonoids there were a high percentage of false positives for inhibition, and a significant number of compounds which increased ThT fluorescence without changing fibrillation kinetics. These findings are consistent with previously reported observations [19, 20].

Because we were able to test as many as 9–14% of the possible flavone derivatives with hydroxyl groups at specific positions, and through comparisons between the strongest inhibitors and flavonoids similar to them, we were able to conclude that, while not additive in their effects, hydroxyflavones with hydroxyl groups at positions 7 and 4’ on the flavone rings are likely to be present in strongly inhibiting flavonoids. Were someone to synthesize hydroxyflavones specifically to try to optimize inhibition of insulin amyloid formation, hydroxyl groups at position 5 and 6 are also somewhat likely candidates for increasing inhibition, while hydroxyl groups at 3 and 3’ should possibly be avoided.

There are a few direct comparisons that can be made between our results and the effects of flavonoids on inhibiting formation of fibrils by proteins other than insulin. Among compounds we studied, we found fewer than 20 studied by methods other than ThT fluorescence and reported as effective inhibitors of different kinds of amyloid fibrils [3, 6, 9–11, 26, 27]. Of these only one was also a strong inhibitor of insulin fibrillation; luteolin (5,7,3’,4’-tetrahydroxyflavone) inhibited formation of human transthyretin fibrils [9]. A study on prion protein (PrP) aggregates found that flavone derivatives effective at preventing amyloid fibre formation in PrP from mouse were ineffective at preventing aggregation in sheep PrP [26]. Combined, these observations indicate that effects of flavonoids may be highly protein specific, sensitive to even relatively small changes in protein sequence.

Further investigations are needed to reveal either more general principles or specific solutions regarding flavone inhibition of amyloid formation. It is likely that, although no single flavone derivative is effective in inhibiting amyloid formation generally, the flavone backbone appears to provide a common base upon which inhibitors can be built for many different amyloids. Thus, the similarities among amyloids find a potentially useful reflection in this family of hydroxyflavones.

## Supporting Information

S1 Fig Example of data fitting and extraction of relative parameters. (PDF)

S2 Fig Example of raw data set of a single batch experiment. (PDF)

S3 Fig Example of simultaneous absorbance and fluorescence experiments. The presented data is an average of 7 repeats.
(PDF)

S1 Table Evaluation of flavones as inhibitors of insulin amyloid-like fibril formation. (PDF)
