# Supplementary figures and images for: Flavone Derivatives as Inhibitors of Insulin Amyloid-Like Fibril Formation
Source: PLoS One. 2015 Mar 23;10(3):e0121231. doi: 10.1371/journal.pone.0121231 (PMC4370379; doi:10.1371/journal.pone.0121231)

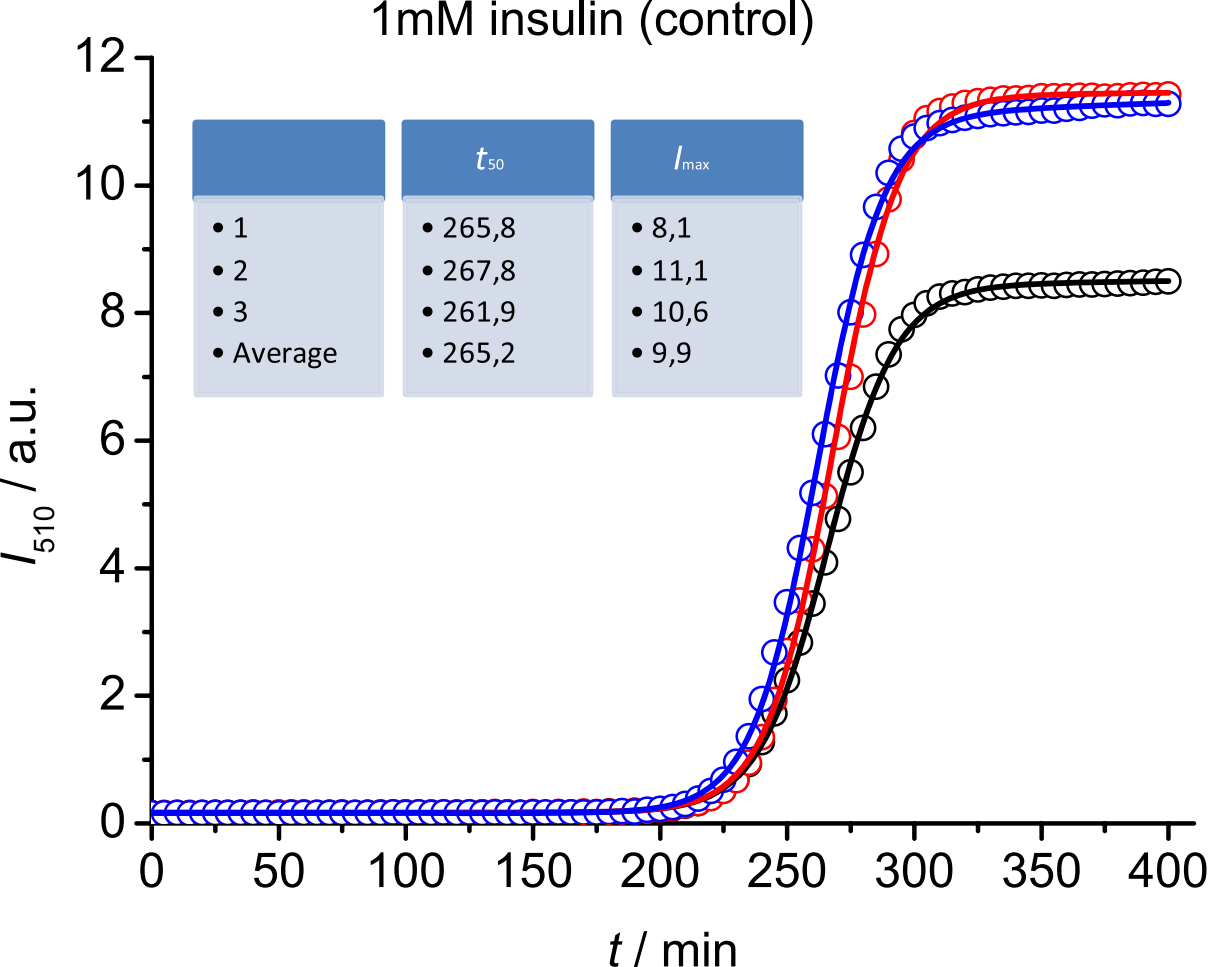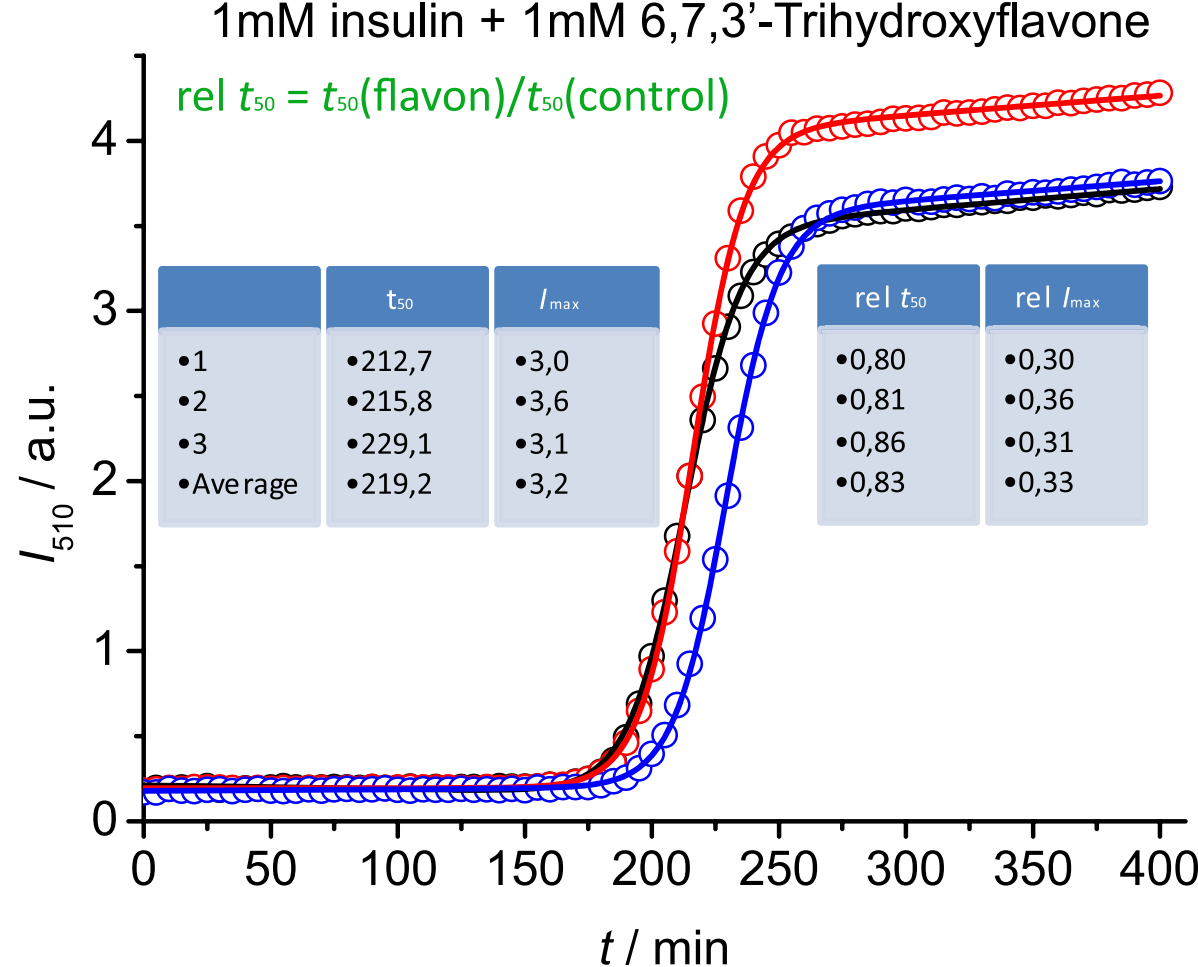

Supplement: S1 Fig — (PDF) [file pone.0121231.s001.pdf]

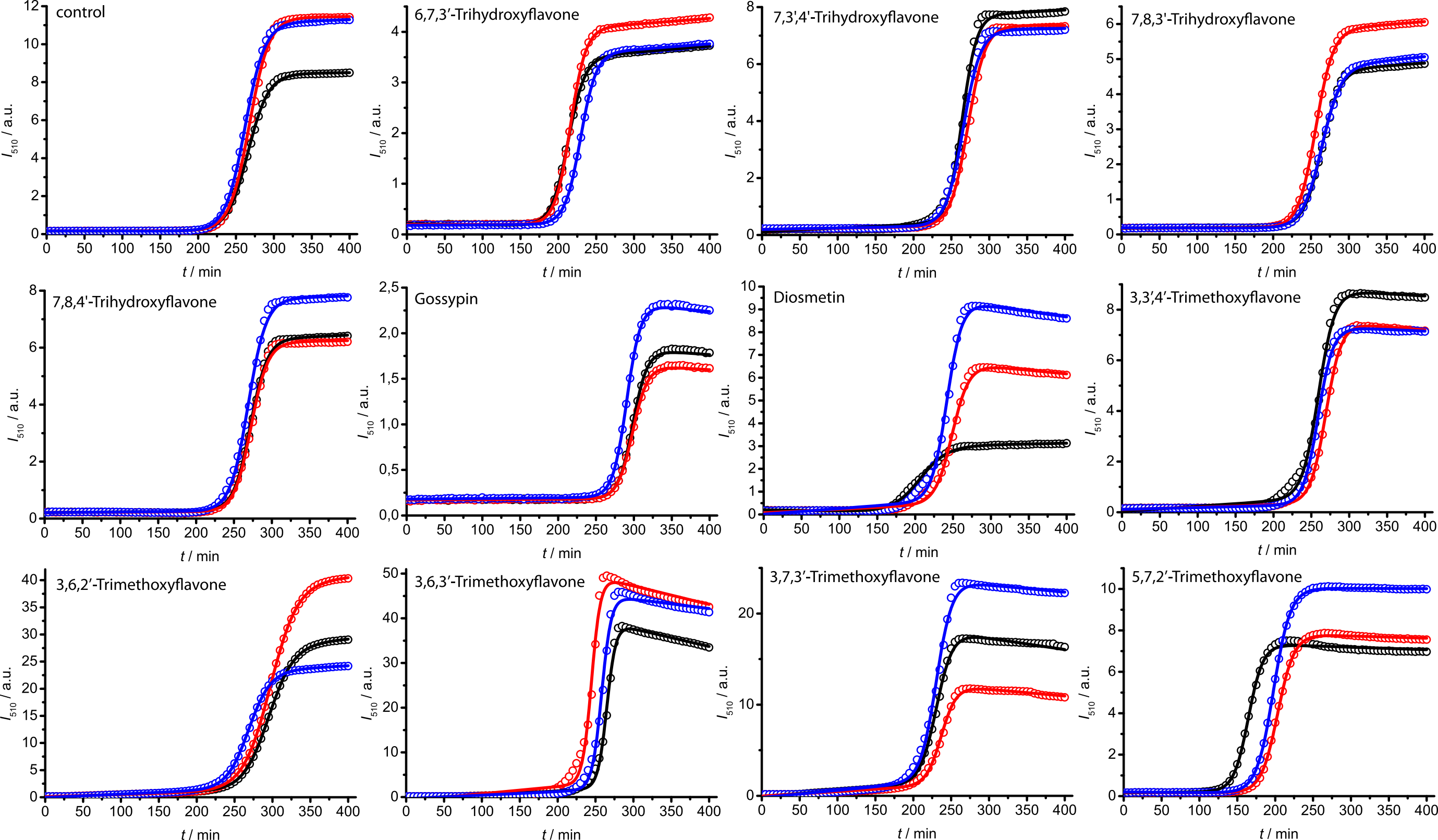

Supplement: S2 Fig — (PDF) [file pone.0121231.s002.pdf]

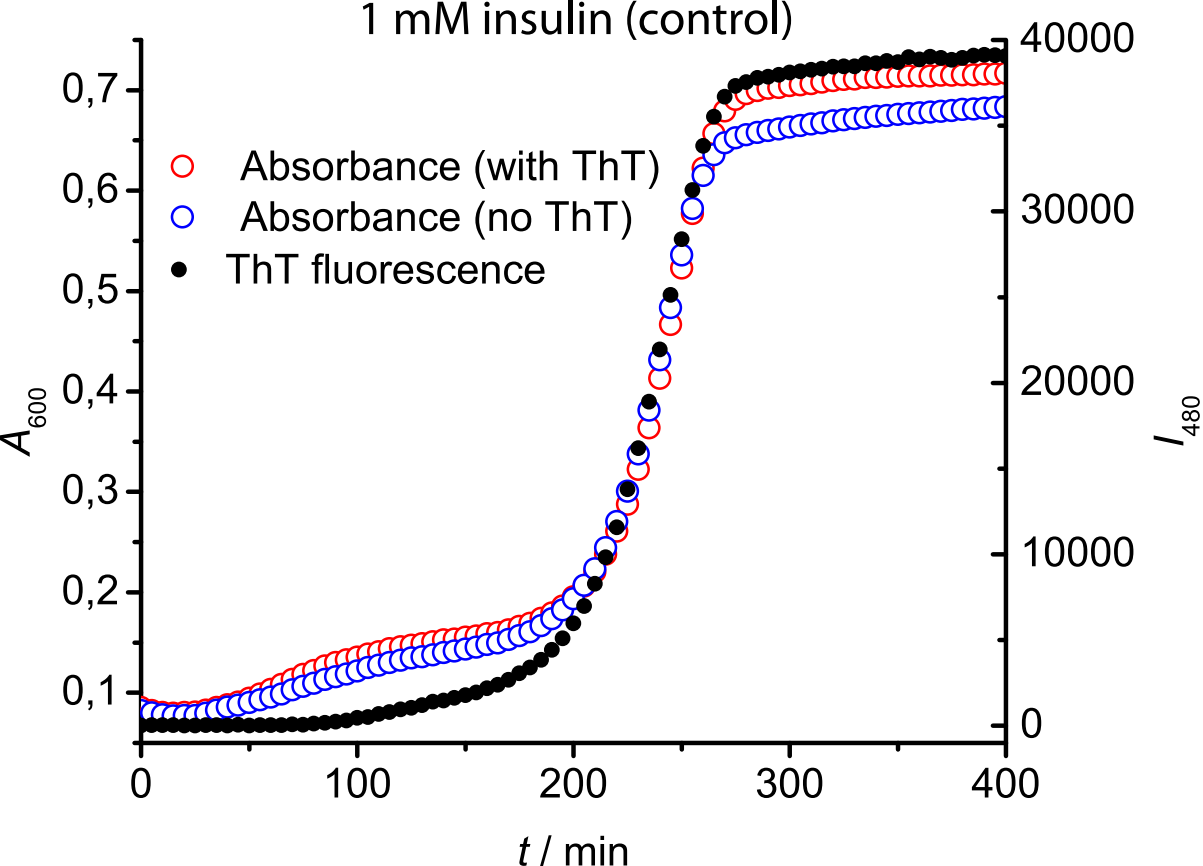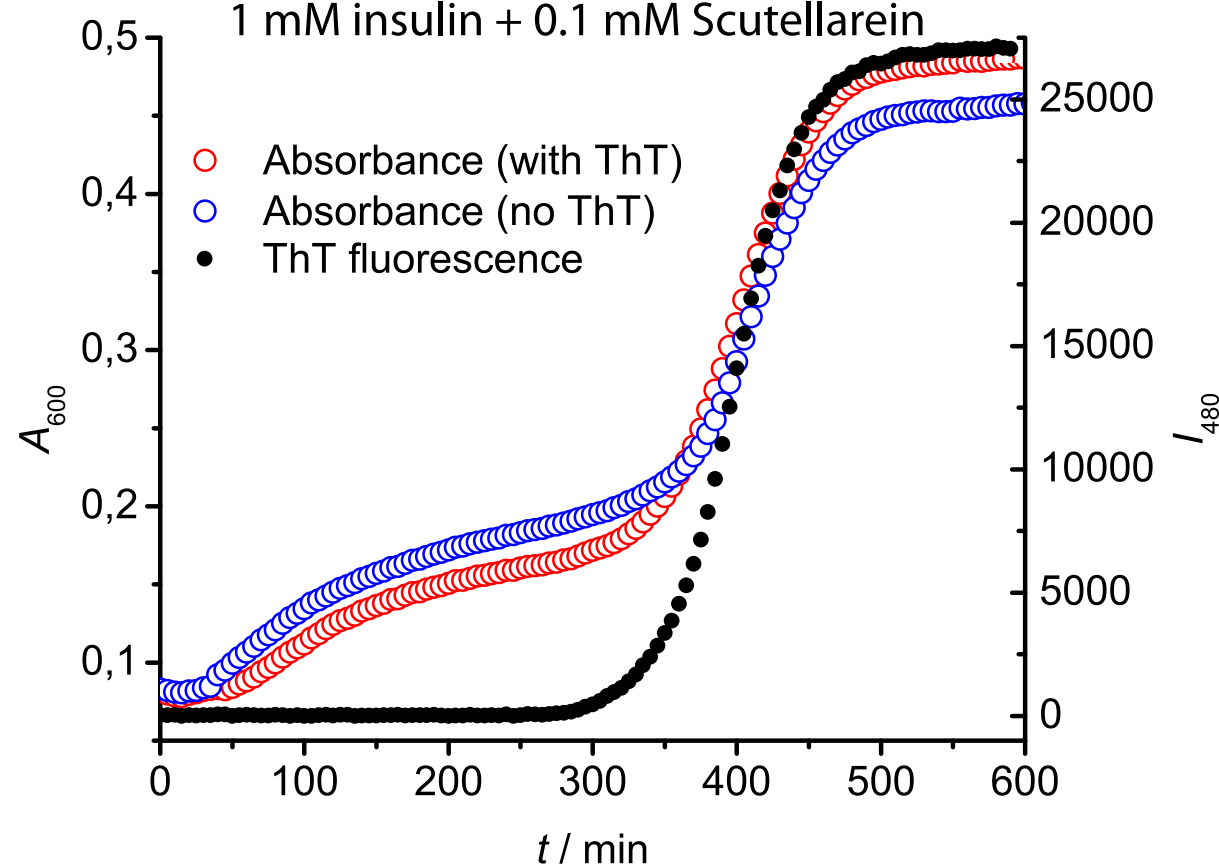

Supplement: S3 Fig — The presented data is an average of 7 repeats. (PDF) [file pone.0121231.s003.pdf]
