# Supplementary material for: Flavone Derivatives as Inhibitors of Insulin Amyloid-Like Fibril Formation
Source: PLoS One. 2015 Mar 23;10(3):e0121231. doi: 10.1371/journal.pone.0121231 (PMC4370379; doi:10.1371/journal.pone.0121231)
Supplement: S1 Table — (PDF) [file pone.0121231.s004.pdf]

Table S1. Evaluation of flavones as inhibiors of insulin amyloid-like fibril formation.

Flavone name	relative t_{50}	error rel. t_{50}	relative I_{max}	error rel. I_{max}
Scutellarein = 5,6,7,4'-Tetrahydroxyflavone	24,80	0,67	0,09	0,01
Luteolin = 5,7,3',4'-Tetrahydroxyflavone	22,35	0,70	0,34	0,11
7,8,2'-Trihydroxyflavone	15,20	1,02	0,27	0,09
3,6,2’,4’,5’-Pentahydroxyflavone	12,35	0,80	0,18	0,09
Gossypetin = 3,5,7,8,3',4'-Hexahydroxyflavone	11,58	0,58	0,02	0,00
Quercetagetin = 3,3',4',5,6,7-Hexahydroxyflavone	2,17	0,38	0,16	0,03
Baicalein = 5,6,7-Trihydroxyflavone	1,87	0,15	0,32	0,07
5,7,2'-Trihydroxyflavone	1,76	0,07	0,69	0,15
3'-Hydroxy-5,6,7,4'-tetramethoxyflavone	1,73	0,07	0,79	0,08
Myricetin = 3,5,7,3',4',5'-Hexahydroxyflavone	1,73	0,10	0,08	0,03
3,5,7-Trihydroxy-3',4',5'-trimethoxyflavone	1,55	0,10	0,65	0,22
7,2’,4’-Trimethoxyflavone	1,43	0,10	0,92	0,14
3',5'-Dimethoxy-3,5,7,4'-tetrahydroxyflavone	1,40	0,11	0,51	0,11
3,6,3’,4’-Tetramethoxyflavone	1,28	0,06	2,15	0,83
3,6,2'-Trihydroxyflavone	1,21	0,05	0,27	0,04
4'-Hydroxy-7-methoxyflavone	1,20	0,04	0,65	0,13
Karanjin = 3-methoxy furano - 2, 3, 7, 8-flavone	1,20	0,09	1,06	0,32
3,6,2',4'-Tetrahydroxyflavone	1,18	0,05	0,42	0,05
7-Hydroxy-4'-methoxyflavone	1,16	0,04	1,57	1,05
6,2’,3’-Trihydroxyflavone	1,15	0,07	0,44	0,04
4'-Hydroxy-5-methoxyflavone	1,15	0,06	0,58	0,20
5,3'-Dihydroxy-6,7,4'-trimethoxyflavone	1,15	0,06	1,06	0,48
3,6-Dimethoxyflavone	1,15	0,08	3,10	1,10
Gossypin = 3,3',4',5,7,8-Hexahydroxyflavone-8-glucoside	1,15	0,08	0,25	0,08
4'-Methoxy-β-naphthoflavone	1,12	0,06	1,67	0,40
3,6,2’-Trimethoxyflavone	1,12	0,10	3,03	0,82
3-Hydroxy-7,8,4’-trimethoxyflavone	1,11	0,07	0,80	0,37
4’-Bromo-6-methylflavone	1,10	0,06	0,85	0,22
3-Hydroxy-6,3’,4’-trimethoxyflavone	1,10	0,02	0,88	0,12
3',4'-Dimethoxy-β-naphthoflavone	1,10	0,04	1,64	0,28
3,6,2’,3’-Tetrahydroxyflavone	1,09	0,01	0,11	0,01
3,6,2’,3’-Tetramethoxyflavone	1,08	0,08	3,04	0,85
Rhamnetin = 3,3',4',5-Tetrahydroxy-7-methoxyflavone	1,08	0,03	0,97	0,34
Galangin = 3,5,7-Trihydroxyflavone	1,08	0,03	0,57	0,16
6,7-Dimethoxyflavone	1,08	0,07	5,21	0,43
6-Hydroxy-7-methoxyflavone	1,08	0,02	0,88	0,13
3,6,3’-Trimethoxyflavone	1,07	0,08	4,47	1,34
5,6-Dihydroxyflavone	1,07	0,04	0,45	0,06

5,2'-Dihydroxyflavone	1,07	0,02	0,78	0,18
Quercetin-3,5,7,3',4'-pentamethyl ether	1,06	0,07	1,07	0,20
8-Bromo-2’,6-dichloroflavone	1,06	0,04	1,19	0,30
5,7-Dimethoxy-3-hydroxyflavone	1,05	0,02	0,68	0,11
5-Hydroxyflavone	1,05	0,06	0,61	0,12
2'-Methoxy-β-naphthoflavone	1,04	0,04	1,87	0,90
Flavone	1,04	0,06	2,39	1,81
3-Hydroxy-6-methoxyflavone	1,04	0,02	0,66	0,25
3,3’,7,8-Tetramethoxyfavone	1,04	0,07	3,99	1,05
5,4'-Dihydroxy-7-methoxyflavone	1,04	0,01	0,62	0,08
Quercetin = 3,5,7,3',4'-Pentahydroxyflavone	1,03	0,02	0,41	0,04
3,3'-Dihydroxyflavone	1,03	0,04	0,44	0,12
7,3’,4’,5’-Tetramethoxyflavone	1,03	0,04	1,47	0,37
2',3'-Dihydroxyflavone	1,03	0,05	0,48	0,13
3-Hydroxy-4’-methoxy-6-methylflavone	1,03	0,06	0,41	0,07
6,4'-Dichloroflavone	1,02	0,04	0,80	0,14
4’-Bromo-6-chloroflavone	1,02	0,07	1,07	0,28
7,8-Dihydroxyflavone	1,02	0,05	0,74	0,33
3,7,3’-Trihydroxyflavone	1,02	0,04	0,55	0,06
6,2’,4’-Trimethoxyflavone	1,02	0,05	1,97	0,45
3,7,3’-Trimethoxyflavone	1,02	0,08	4,69	2,48
3,4'-Dihydroxyflavone	1,02	0,08	1,93	0,28
7,3'-Dimethoxyflavone	1,02	0,07	1,28	0,32
7,2'-Dihydroxyflavone	1,01	0,03	0,77	0,08
4’-Bromo-6-chloro-7-methylflavone	1,01	0,03	1,01	0,08
6-Bromo-2’-chloroflavone	1,01	0,05	1,33	0,34
Acacetin = 5,7-Dihydroxy-4'-methoxyflavone	1,00	0,03	0,97	0,15
6,4’-Dibromoflavone	1,00	0,03	0,66	0,19
2’,6’-Dichloro-7-methylflavone	1,00	0,01	0,98	0,22
3,6,3'-Trihydroxyflavone	1,00	0,04	0,69	0,44
4'-Hydroxy-3'-methoxyflavone	1,00	0,09	0,68	0,25
7,8-Dimethoxyflavone	0,99	0,05	2,40	1,33
3,7,8,2’-Tetrahydroxyflavone	0,99	0,02	0,57	0,10
6,4'-Dihydroxyflavone	0,99	0,04	0,84	0,15
7-Hydroxy-2’-methoxyflavone	0,99	0,02	0,87	0,14
3-Hydroxy-7,8,2’-trimethoxyflavone	0,99	0,01	0,75	0,17
6,4’-Dibromoflavone	0,99	0,02	0,69	0,13
Homoorientin = Luteolin-6-C-Glucoside	0,99	0,04	0,85	0,29
Kaempferide = 4’-Methoxy-3,5,7-trihydroxyflavone	0,99	0,03	0,46	0,12
4’-Chloro-6-methylflavone	0,99	0,03	0,93	0,19
7-Hydroxy-5-methylflavone	0,99	0,03	0,76	0,10

2’-Bromo-6-chloroflavone	0,99	0,03	1,38	0,29
3’-Methoxy-8-methylflavone	0,99	0,06	3,42	1,69
6,4’-Dimethoxy-3-hydroxyflavone	0,99	0,03	0,84	0,25
3-Hydroxy-4'-methoxyflavone	0,99	0,02	0,99	0,20
6,8-Dichloroflavone	0,99	0,01	0,73	0,14
6,2'-Dichloroflavone	0,99	0,01	0,83	0,11
4'-Hydroxyflavone	0,99	0,01	0,82	0,26
6,8,4’-Trichloroflavone	0,98	0,02	0,78	0,12
Kaempferol = 3,5,7,4'-Tetrahydroxyflavone	0,98	0,05	0,89	0,17
3,7,8,4’-Tetramethoxyflavone	0,98	0,09	2,09	0,83
7,3',4'-Trihydroxyflavone	0,98	0,08	0,69	0,04
4’-Chloro-6,8-dibromoflavone	0,98	0,03	0,73	0,10
6-Bromoflavone	0,98	0,04	0,93	0,18
6-Chloro-7-methylflavone	0,98	0,02	0,68	0,16
3,6,4'-Trihydroxyflavone	0,98	0,02	0,44	0,04
2',4'-Dihydroxyflavone	0,98	0,04	0,56	0,16
3',4'-Dihydroxy-β-naphthoflavone	0,98	0,07	0,93	0,37
6,8-Dichloro-4’-methylflavone	0,98	0,02	0,67	0,09
6,7-Dihydroxyflavone	0,98	0,06	1,16	0,19
4'-Methoxyflavonol, 4'-Methoxy-3-hydroxyflavone	0,98	0,05	1,03	0,17
6,3',4'-Trihydroxyflavone	0,98	0,03	0,62	0,21
3-Hydroxy-2’,4’,5’-trimethoxyflavone	0,97	0,02	0,87	0,10
4’-Methylflavone	0,97	0,04	1,45	0,91
3,7-Dihydroxyflavone	0,97	0,04	0,79	0,14
3,2'-Dimethoxyflavone	0,97	0,03	1,35	0,37
8-Bromo-6-chloro-2’,5’-dimethoxyflavone	0,97	0,03	1,53	0,46
3’,4’-Dimethoxy-3-hydroxy-6-methylflavone	0,97	0,01	0,76	0,12
6-Bromo-2’-methylflavone	0,97	0,06	1,04	0,27
7,8,4'-Trihydroxyflavone	0,97	0,04	0,80	0,18
5,7,3’,4’,5’-Pentahydroxyflavone	0,97	0,05	1,02	0,17
6-Chloro-7,2’-dimethylflavone	0,97	0,03	1,74	1,44
7,2'-Dimethoxyflavone	0,97	0,05	1,04	0,32
3-Hydroxy-2'-methoxyflavone	0,97	0,07	0,75	0,22
5-Hydroxy-7-methoxyflavone	0,97	0,02	0,52	0,08
3-Hydroxy-7,2',4'-trimethoxyflavone	0,97	0,03	0,90	0,18
7,8,3'-Trihydroxyflavone	0,97	0,03	0,73	0,20
3-Hydroxy-7,8,2’,3’-tetramethoxyflavone	0,96	0,02	1,05	0,12
3-Hydroxy-7,3’,4’,5’-tetramethoxyflavone	0,96	0,03	0,69	0,09
6,4'-Dichloro-7-methylflavone	0,96	0,03	0,80	0,14
6,3'-Dihydroxyflavone	0,96	0,04	0,71	0,12
6-Bromo-4’-methylflavone	0,96	0,01	0,59	0,15

3,4'-Dimethoxy-5,7,3'-trihydroxyflavone	0,96	0,05	1,95	0,40
Apigenin = 5,7,4'-Trihydroxyflavone	0,96	0,04	0,53	0,07
7,3’,4’,5’-Tetrahydroxyflavone	0,96	0,05	0,90	0,26
3-Hydroxy-7,2’,3’-trimethoxyflavone	0,96	0,02	1,05	0,23
3-Hydroxy-6-methylflavone	0,96	0,04	0,51	0,08
3,5-Dihydroxyflavone	0,96	0,02	0,68	0,23
6-Chloro-7,4’-dimethylflavone	0,96	0,02	0,79	0,24
2’,4’-Dimethoxy-3-hydroxyflavone	0,96	0,03	0,98	0,11
6-Chloro-4’-methylflavone	0,96	0,02	0,54	0,12
3,3’,4’-Trimethoxyflavone	0,96	0,06	0,97	0,30
3,2’,4’,5’-Tetramethoxyflavone	0,96	0,01	1,73	0,60
2’,3’-Dimethoxy-3-hydroxyflavone	0,96	0,03	0,72	0,13
3,3',4'-Trihydroxyflavone	0,95	0,01	0,44	0,08
4'-Hydroxy-α-naphthoflavone	0,95	0,03	1,12	0,19
6,8,2’-Trichloroflavone	0,95	0,02	0,78	0,12
3-Hydroxy-6,2',4'-trimethoxyflavone	0,95	0,02	0,94	0,25
Morin = 3,5,7,2',4'-Pentahydroxyflavone	0,95	0,06	0,31	0,10
6-Hydroxy-3'-methoxyflavone	0,94	0,01	0,76	0,06
6,2’-Dimethoxy-3-hydroxyflavone	0,94	0,04	0,97	0,23
6-Bromo-3’-methylflavone	0,94	0,02	0,84	0,20
4'-Methoxy-6-methylflavone	0,94	0,06	0,74	0,13
7,3'-Dihydroxyflavone	0,94	0,06	1,06	0,20
3'-Hydroxy-β-naphthoflavone	0,94	0,07	1,46	0,18
3'-Hydroxyflavone	0,94	0,03	0,76	0,12
2'-Hydroxy-β-naphthoflavone	0,94	0,03	0,85	0,10
3,7,2’,4’,5’-Pentamethoxyflavone	0,94	0,04	1,53	0,31
6-Chloroflavone	0,94	0,02	0,72	0,18
3,7,8,2’-Tetramethoxyflavone	0,94	0,02	3,01	0,94
2’,4’-Dimethoxy-3-hydroxy-6-methylflavone	0,93	0,04	0,94	0,18
6-Methoxyflavone	0,93	0,04	0,88	0,21
3-Hydroxy-2’,4’,5’,6-tetramethoxy flavone	0,93	0,02	1,25	0,22
6,4'-Dimethoxyflavone	0,93	0,06	1,35	0,49
7-Hydroxyflavone	0,93	0,05	0,93	0,38
6-Hydroxy-4’-methoxyflavone	0,93	0,03	0,54	0,05
6,8-Dibromoflavone	0,93	0,02	0,69	0,12
3-Hydroxy-7-methoxyflavone	0,93	0,03	0,72	0,20
7,2’-Dimethoxy-3-hydroxyflavone	0,93	0,03	1,08	0,19
5-Hydroxy-3'-methoxyflavone	0,93	0,02	0,83	0,20
7,8,3',4'-Tetrahydroxyflavone	0,93	0,07	0,40	0,12
4’-Chloroflavone	0,93	0,03	0,70	0,18
3-Hydroxy-3’,4’,5’-trimethoxyflavone	0,93	0,02	1,17	0,22

5,4'-Dihydroxyflavone	0,93	0,01	0,38	0,06
6,2'-Dihydroxyflavone	0,93	0,01	0,52	0,08
6,3’-Dimethoxy-3-hydroxyflavone	0,93	0,02	0,88	0,07
3,7,3’,4’,5’-Pentahydroxyflavone	0,93	0,06	0,41	0,07
5,3',4'-Trihydroxyflavone	0,93	0,02	1,27	0,33
8-Methylflavone	0,93	0,02	0,54	0,09
6,8-Dichloro-2’-methylflavone	0,93	0,04	0,96	0,12
4'-Methoxy-8-methylflavone	0,92	0,07	2,64	0,65
7-Hydroxy-3-methylflavone	0,92	0,04	0,80	0,11
6-Bromo-4’-chloroflavone	0,92	0,04	0,64	0,19
3-Hydroxy-7-methoxyflavone	0,92	0,02	0,73	0,13
6,3’-Dimethylflavone	0,92	0,02	1,31	0,33
2',3'-Dimethoxyflavone	0,92	0,03	1,09	0,19
6,2’-Dimethylflavone	0,92	0,03	1,35	0,33
3-Hydroxy-6,2',3'-trimethoxyflavone	0,91	0,02	0,69	0,12
7,8,3',4'-Tetramethoxyflavone	0,91	0,02	0,85	0,14
7-Hydroxy-3'-methoxyflavone	0,91	0,01	1,01	0,29
3'-Benzyloxy-5,6,7,4'-tetramethoxyflavone	0,91	0,05	3,42	1,52
6-Chloro-7,3’-dimethylflavone	0,91	0,02	1,05	0,18
5,6-Dihydroxy-7-methoxyflavone	0,90	0,04	2,15	0,86
8-Hydroxy-7-methoxyflavone	0,90	0,03	0,74	0,19
Diosmetin = 5,7,3'-Trihydroxy-4'-methoxyflavone	0,90	0,07	0,70	0,23
Baicalein-5,6,7-trimethylether	0,90	0,05	1,43	0,20
2’-Chloro-6-methylflavone	0,90	0,04	1,50	0,36
3,2'-Dihydroxyflavone	0,90	0,02	0,29	0,06
Gardenin = 5-Hydroxy-3',4',5',6,7,8-hexamethoxyflavone	0,90	0,04	1,92	0,41
4’-Benzyloxy-5,7-dimethoxyflavone	0,90	0,06	2,25	0,71
2'-Hydroxyflavone	0,90	0,03	1,09	0,31
6-Chloro-2’-methylflavone	0,90	0,02	1,91	0,43
5,7-Dihydroxyflavone	0,90	0,06	0,73	0,10
3-Hydroxy-3'-methoxyflavone	0,90	0,06	0,61	0,16
3,7,3’,4’,5’-Pentamethoxyflavone	0,89	0,03	1,74	0,38
6-Hydroxy-2'-methoxyflavone	0,89	0,01	0,95	0,17
5,3'-Dihydroxyflavone	0,88	0,02	0,52	0,08
Kaempferol-3,7,4'-trimethyl ether	0,88	0,03	0,63	0,10
Tangeretin = 4’,5,6,7,8-Pentamethoxyflavone	0,88	0,02	1,00	0,15
6,7,3’-Trihydroxyflavone	0,88	0,05	0,61	0,11
3-Methoxyflavone	0,88	0,02	2,38	0,78
7,3’-Dimethoxy-3-hydroxyflavone	0,88	0,02	0,74	0,18
4’-Hydroxy-6-methylflavone	0,87	0,02	0,49	0,11
3-Hydroxy-5-methoxyflavone	0,87	0,07	3,06	1,14

3,7-Dimethoxyflavone	0,87	0,02	0,91	0,25
7,4’-Dimethoxy-3-hydroxyflavone	0,87	0,01	0,84	0,24
7,4'-Dihydroxyflavone	0,87	0,04	1,32	0,53
3’,5’-Dihydroxyflavone	0,87	0,04	0,84	0,15
3,7,4'-Trihydroxyflavone	0,87	0,03	0,73	0,26
8-Carboxy-3-methylflavone	0,87	0,01	0,93	0,16
3',4'-Dihydroxyflavone	0,86	0,07	0,50	0,04
6,7,3’,4’-Tetramethoxyflavone	0,86	0,06	1,74	0,13
3',4'-Dimethoxy-α-naphthoflavone	0,85	0,07	1,90	0,56
3,6-Dihydroxyflavone	0,85	0,03	0,57	0,08
5,7,3’,4’,5’-Pentamethoxyflavone	0,85	0,05	1,27	0,27
4'-Hydroxy-6-methoxyflavone	0,85	0,05	0,48	0,10
3,2’,4’,5’,6-Pentamethoxyflavone	0,85	0,03	4,57	1,20
5,7,3’-Trimethoxyflavone	0,85	0,06	1,60	0,87
3’-Hydroxy-6-methylflavone	0,84	0,02	0,68	0,12
3-Hydroxy-3',4'-Dimethoxyflavone	0,84	0,03	1,61	0,40
6-Hydroxyflavone	0,84	0,01	0,55	0,09
6-Chloro-2’,5’-dimethoxy-7-methylflavone	0,84	0,05	2,32	0,51
6-Methylflavone	0,83	0,06	2,42	0,48
6,2’,3’-Trimethoxyflavone	0,83	0,06	2,17	0,58
6,3'-Dimethoxyflavone	0,83	0,04	2,44	1,08
3'-Methoxyflavone	0,82	0,03	2,49	0,96
4'-Methoxyflavone	0,82	0,05	0,91	0,39
3,7-Dihydroxy-3',4'-dimethoxyflavone	0,82	0,07	0,30	0,04
5,7,2’-Trimethoxyflavone	0,82	0,07	0,95	0,33
2’-Chloroflavone	0,81	0,02	1,18	0,36
4’-Methoxy-α-naphthoflavone	0,81	0,03	1,38	0,20
2'-Methoxyflavone	0,81	0,07	3,58	0,46
5,7,3',4'-Tetramethoxyflavone	0,80	0,03	1,27	0,32
6,7,3’,4’-Tetrahydroxyflavone	0,80	0,05	0,25	0,07
2'-Methoxy-α-naphthoflavone	0,80	0,07	2,65	0,51
3-Hydroxyflavone	0,79	0,07	1,56	0,33
5,7-Dimethoxyflavone	0,79	0,06	1,48	0,13
3,4'-Dimethoxyflavone	0,79	0,03	1,63	0,21
5,2'-Dimethoxyflavone	0,79	0,03	2,11	0,72
5,7,4'-Trimethoxyflavone	0,77	0,04	0,60	0,11
3'-Hydroxy-α-naphthoflavone	0,77	0,07	2,52	0,25
2'-Hydroxy-α-naphthoflavone	0,75	0,06	1,88	0,21
3-Hydroxy-7,8,3’-trimethoxyflavone	0,75	0,03	1,53	0,16
7,4'-Dimethoxyflavone	0,75	0,04	0,70	0,15
6-Methoxyluteolin = 6-methoxy 5,7,3',4'-tetrahydroxyflavone	0,75	0,02	0,12	0,03

3-Hydroxy-6-methyl-3’,4’-methylenedioxy flavone	0,74	0,06	1,91	0,26
3,6,3’,4’-Tetrahydroxyflavone	0,72	0,01	0,34	0,05
7,3',4'-Trimethoxyflavone	0,72	0,06	0,89	0,36
2’-Methoxy-6-methylflavone	0,72	0,03	2,29	0,87
7,2’,3’-Trimethoxyflavone	0,72	0,04	1,26	0,32
Quercetin-3,7,3',4'-tetramethyl ether	0,71	0,06	0,66	0,13
3',4'-Dihydroxy-α-naphthoflavone	0,70	0,06	2,00	0,78
5,6,7,3’,4’-Pentamethoxyflavone	0,69	0,07	0,55	0,19
7-Methoxyflavone	0,69	0,05	2,41	0,77
3',4'-Dimethoxyflavone	0,69	0,06	0,55	0,07
Scutellarein tetramethyl ether	0,67	0,02	0,59	0,04
5-Methoxyflavone	0,65	0,04	1,47	0,19
2',4'-Dimethoxyflavone	0,64	0,05	3,64	0,90
Fisetin = 3,7,3',4'-Tetrahydroxyflavone	0,62	0,03	0,97	0,14
5,4'-Dimethoxyflavone	0,61	0,04	1,43	0,44
5,3’-Dimethoxyflavone	0,61	0,03	1,24	0,60
3,3'-Dimethoxyflavone	0,60	0,03	5,21	0,69
6,2'-Dimethoxyflavone	0,54	0,02	2,32	0,58
4'-Hydroxy-β-naphthoflavone	0,54	0,02	1,43	0,39
2’,5’-Dimethoxyflavone	0,53	0,01	1,95	0,56
2’,5’-Dimethoxy-6-fluoroflavone	0,46	0,04	2,10	0,32
3’-Methoxy-6-methylflavone	0,45	0,03	1,42	0,16
